# Molecular epidemiology and phylogenetic analysis of influenza viruses A (H3N2) and B/Victoria during the COVID-19 pandemic in Guangdong, China

**DOI:** 10.1186/s40249-024-01218-z

**Published:** 2024-08-01

**Authors:** Zhiqi Zeng, Yong Liu, Wenxiang Jin, Jingyi Liang, Jinbin Chen, Ruihan Chen, Qianying Li, Wenda Guan, Lixi Liang, Qiubao Wu, Yuanfang Lai, Xiaoyan Deng, Zhengshi Lin, Chitin Hon, Zifeng Yang

**Affiliations:** 1 State Key Laboratory of Respiratory Disease, National Clinical Research Center for Respiratory Disease, Guangzhou Institute of Respiratory Health, the First Affiliated Hospital of Guangzhou Medical University, Guangzhou, Guangdong 510180 P.R. China; 2 Guangzhou Key Laboratory for Clinical Rapid Diagnosis and Early Warning of Infectious Diseases, KingMed School of Laboratory Medicine, Guangzhou Medical University, Guangzhou, People’s Republic of China; 3 Kingmed Virology Diagnostic and Translational Center, Guangzhou Kingmed Center for Clinical Laboratory Co., Ltd., Guangzhou, China; 4 Department of Engineering Science, Faculty of Innovation Engineering, Macau University of Science and Technology, Taipa, Macau China; 5 Guangzhou Laboratory, Guangzhou, China; 6 Respiratory Disease AI Laboratory on Epidemic Intelligence and Medical Big Data Instrument Applications, Faculty of Innovative Engineering, Macau University of Science and Technology, Macau SAR, China

**Keywords:** Insidious transmission, Influenza, COVID-19 control, Genetic diversity, Evolution

## Abstract

**Background:**

Non-pharmaceutical measures and travel restrictions have halted the spread of coronavirus disease 2019 (COVID-19) and influenza. Nonetheless, with COVID-19 restrictions lifted, an unanticipated outbreak of the influenza B/Victoria virus in late 2021 and another influenza H3N2 outbreak in mid-2022 occurred in Guangdong, southern China. The mechanism underlying this phenomenon remains unknown. To better prepare for potential influenza outbreaks during COVID-19 pandemic, we studied the molecular epidemiology and phylogenetics of influenza A(H3N2) and B/Victoria that circulated during the COVID-19 pandemic in this region.

**Methods:**

From January 1, 2018 to December 31, 2022, we collected throat swabs from 173,401 patients in Guangdong who had acute respiratory tract infections. Influenza viruses in the samples were tested using reverse transcription-polymerase chain reaction, followed by subtype identification and sequencing of hemagglutinin (HA) and neuraminidase (NA) genes. Phylogenetic and genetic diversity analyses were performed on both genes from 403 samples. A rigorous molecular clock was aligned with the phylogenetic tree to measure the rate of viral evolution and the root-to-tip distance within strains in different years was assessed using regression curve models to determine the correlation.

**Results:**

During the early period of COVID-19 control, various influenza viruses were nearly undetectable in respiratory specimens. When control measures were relaxed in January 2020, the influenza infection rate peaked at 4.94% (39/789) in December 2021, with the influenza B/Victoria accounting for 87.18% (34/39) of the total influenza cases. Six months later, the influenza infection rate again increased and peaked at 11.34% (255/2248) in June 2022; influenza A/H3N2 accounted for 94.51% (241/255) of the total influenza cases in autumn 2022. The diverse geographic distribution of HA genes of B/Victoria and A/H3N2 had drastically reduced, and most strains originated from China. The rate of B/Victoria HA evolution (3.11 × 10^−3^, *P* < 0.05) was 1.7 times faster than before the COVID-19 outbreak (1.80 × 10^−3^, *P* < 0.05). Likewise, the H3N2 HA gene’s evolution rate was 7.96 × 10^−3^ (*P* < 0.05), which is 2.1 times faster than the strains’ pre-COVID-19 evolution rate (3.81 × 10^−3^, *P* < 0.05).

**Conclusions:**

Despite the extraordinarily low detection rate of influenza infection, concealed influenza transmission may occur between individuals during strict COVID-19 control. This ultimately leads to the accumulation of viral mutations and accelerated evolution of H3N2 and B/Victoria viruses. Monitoring the evolution of influenza may provide insights and alerts regarding potential epidemics in the future.

**Supplementary Information:**

The online version contains supplementary material available at 10.1186/s40249-024-01218-z.

## Background

Seasonal influenza is a serious public health issue around the world, impacting 5–15% of the population and causing roughly half a million fatalities each year [1, 2]. As a result, preventing seasonal influenza is a top worldwide health concern [3, 4]. In recent years, most cases of seasonal influenza have been caused by the simultaneous spread of influenza A and B viruses [5]. In temperate climates, influenza typically occurs once a year during the cold season; however, year-round influenza activity can be seen in tropical and subtropical areas with varied timing and duration [6]. Influenza transmission patterns in China are complex and depend on latitude, geographic region, and viral strain [7]. The intricate epidemiological nature of influenza makes it challenging to predict future influenza outbreaks in a timely manner.

The coronavirus disease 2019 (COVID-19) pandemic, caused by severe acute respiratory syndrome coronavirus 2 (SARS-CoV-2), emerged in early 2020 and rapidly spread worldwide. Non-pharmacological interventions (NPIs), which include travel restrictions, wearing masks, physical distancing, and staying at home, were implemented globally to curb SARS-CoV-2 transmission. NPIs also reduced the transmission of other respiratory viruses, such as influenza [8]. For example, influenza activity decreased substantially during March 2020 in the United States, reaching an all-time low in the summer of 2020, and the low influenza activity remained from October 2020 to May 2021 [9]. The same phenomenon was also reported in Australia, Chile, and South Africa, where influenza activity was exceptionally low during the regular influenza season in the Southern Hemisphere [9–12]. Similarly, influenza activity in China decreased significantly during the COVID-19 pandemic; however, it gradually increased in 2021, accompanied by a shift in circulating virus, from the influenza A subtype to the influenza B/Victoria subtype, and the absence of the B/Yamagata lineage [12, 13]. Currently, it remains unclear how human activity changes the regular transmission pattern of influenza virus and influences viral evolution. To answer this question, we selected Guangdong Province, which has a subtropical monsoon climate and a permanent population of 126 million as of 2021. We collected respiratory samples from patients with acute respiratory tract infections (ARTI) from 2018 to 2022 across the province. We studied the phylogenetics and genetic diversity of influenza A(H3N2) and B/Victoria. Our findings suggest that insidious influenza virus transmission and evolutionary alterations may have occurred during the COVID-19 pandemic.

## Methods

### Sample collection

Throat swabs were obtained from 173,401 patients with ARTI in Guangdong Province between January 2018 (pre-pandemic) and December 2022 (mid-pandemic). We only included samples from individuals with influenza-like symptoms (fever 37.3 ℃) accompanied by at least one respiratory symptom (sore throat, cough, coryza, or shortness of breath) and whose physician had prescribed an influenza virus laboratory test (Fig. S1). Throat swab samples were chilled at 2–8 ℃ in viral transport medium and transported to Kingmed Diagnostics (KMD) laboratory. Samples were either examined immediately or stored at − 80 ℃ until testing. KMD is a China-based accredited commercial medical laboratory.

### Influenza detection and Sanger sequencing

Viral RNA/DNA was extracted from the swabs using nucleic acid extraction kits (Shanghai Kehua Bio-Engineering Co., Ltd., Shanghai, China). TaqMan real-time polymerase chain reaction kits (ZJ Bio-Tech, Shanghai, China) were used to test the influenza A and B viruses. Influenza-positive samples were genotyped for influenza A H1/H3 and influenza B Yamagata/Victoria subtypes (test kits from Baso, Guangdong, China). Amplification conditions for the reverse transcription (RT) step were 1 cycle at 50 ℃ for 30 min and 1 cycle at 94 ℃ for 2 min, followed by 35 cycles at 94 ℃ for 30 s, 50 ℃ for 30 s and 72℃ for 1 kb/min, with a final extension step at 72℃ for 10 min. All amplifications were performed in a thermal cycler (Bio-Rad, Hercules, CA, USA). Sequences of RT-PCR primers are listed in Table S1. In total, 207 influenza A-positive samples and 196 influenza B-positive samples were sent to IGE Biotechnology, Ltd. (Guangzhou, China) for Sanger sequencing of hemagglutinin (HA) and neuraminidase (NA) genes.

### Bioinformatics analysis

Gene clustering was analyzed using CD-HIT software (http://weizhongli-lab.org/cd-hit). Identical nucleotide sequences were grouped together. For genetic diversity analysis, we downloaded the HA and NA gene sequences of H3N2 and B/Victoria influenza viruses collected in Africa, Antarctica, Asia, Europe, North America, Oceania, and South America during January 2019 to December 2022 and listed in the GISAID database (https://www.gisaid.org). Sequence multiple alignment was performed using MUSCLE software (http://www.drive5.com/muscle/manual) with default parameters. IQ-TREE (http://www.iqtree.org) software was used to build the phylogenetic tree using the maximum likelihood method. The bootstrap was set to 1000 times, and the FLU + G4 model was used for the substitution model. The ratio of the number of non-synonymous to synonymous (dN/dS) mutations was estimated using the single-likelihood ancestor counting method in DataMonkey (http://www.datamonkey.org). The mixed effects model of evolution in DataMonkey was used to identify codon sites undergoing positive selection, with a significance level of *P* < 0.1. The phylogenetic trees were plotted using the R version 4.2.0 (R Foundation for Statistical Computing, Vienna, Austria). Using the R “treedater” package, a strict molecular clock was matched to the phylogenetic tree to estimate the time of common ancestry and evolutionary rate.

### Statistical analyses

We conducted statistical analysis using R version 4.2.0 (R Foundation for Statistical Computing, Vienna, Austria). The root-to-tip distance within strains in different years was assessed using regression curve models to determine the correlation. Likelihood ratio testing was used to examine the significance of specific mutation sites under episodic diversifying positive selection. *P* value < 0.05 was considered significant.

## Results

**Fig. 1:**
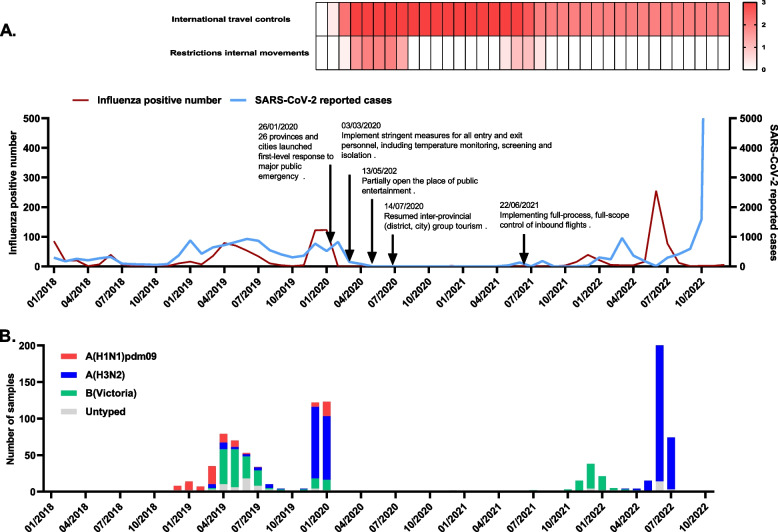
Epidemiology of seasonal influenza virus between 2018 and 2022. **A** Monthly detection of influenza virus and SARS-CoV-2 from January 2018 to December 2022. The red line shows positive cases of influenza virus. Blue lines represent the number of new SARS-CoV-2 cases, according to statistics from the Guangdong Health Commission (http://cdcp.gd.gov.cn/); the heat map at the top right is based on the Oxford COVID-19 Government Response Tracker (OxCGRT) database, reflecting the changes in internal movement restrictions and international travel controls. Arrows highlight the COVID-19 epidemic control policy in Guangdong Province, China. Grading of restrictions on internal movement: 0-no measures; 1-recommend not traveling between regions/cities; 2-internal movement restrictions in place. Grading of international travel controls: 0-no measures; 1-screening; 2-quarantine arrivals from high-risk regions; 3-ban on arrivals from some regions. **B** Number of influenza virus subtypes. Commercial kits detected 1012 influenza-positive samples during 2018–2022: H1N1 (red), H3N2 (blue), Victoria (green), and Untyped (grey). “Untyped”: the test was not done because of too little remaining sample

Epidemic of seasonal influenza in the Guangdong of China from 2020 to 2022. From January 2018 to December 2022, 173,401 throat swabs were collected from patients with ARTI. Among them 132,031 (76.14%) were outpatients from 262 hospitals or health institutions in 21 cities: Guangzhou, Shenzhen, Foshan, Dongguan, Zhongshan, Zhuhai, Jiangmen, Zhaoqing, Huizhou, Shantou, Chaozhou, Jieyang, Shanwei, Zhejiang, Maoming, Yangjiang, Shaoguan, Qingyuan, Yunfu, Meizhou, and Heyuan. Distribution of samples in clinical department was displayed in Table.S2. A total of 1216 samples finally tested positive for influenza virus (Table.S3, Fig. S1). Figure 1A displays the time of changes in influenza cases during this period; control measures were collected from official websites [14–18]. Before the COVID-19 pandemic, there were four spikes in influenza cases: winter 2017–2018, fall 2018, winter 2018–2019, and winter 2019–2020. Influenza A/H1N1 infection was prevalent in autumn 2018, but switched to influenza B/Victoria in winter 2018–2019 (Fig. 1B, Table S2). In the following winter (2019–2020), influenza cases soared again, reaching a new high (47.67%, 123/258), with most patients infected with influenza A/H3N2. The COVID-19 pandemic began around this period. In January 2020, due to NPIs execution, including cancelling public events and restrictions on people movement and gathering, very few samples tested influenza virus positive (0.13%, 8/6256) from February 2020 to October 2021 in Guangdong Province. Regardless intermittent relaxation of the NPIs (e.g. resuming inter-provincial group tourism and restaurant dine-in services), influenza cases still remained at a very low level during the winter of 2020–2021. Nonetheless, In the winter of 2021–2022, the number of influenza cases increased gradually, peaking at 39 cases per month (4.94%, 39/789). Of the total number of influenza cases, influenza B Victoria accounted for 87.18% (34/39) of cases. During the summer of 2022, despite the prohibition on international travel, the number of influenza cases doubled (11.34%, 255/2248) compared to the previous influenza spike (4.94%, 39/789). The most common virus, influenza A H3N2, accounted for 94.51% (241/255) of the total influenza cases (Fig. 1A, B).

### Genetic characterization of B/Victoria and A/H3N2 influenza viruses

**Fig. 2:**
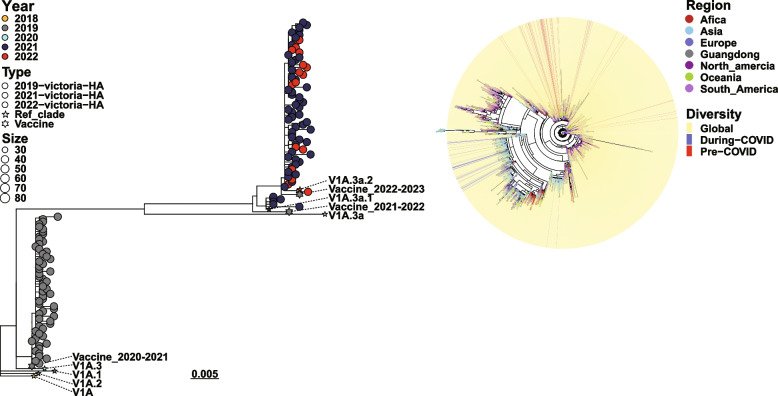
Phylogenic tree of the HA gene of B/Victoria influenza viruses during 2019–2022. **A** Phylogenetic tree of the HA gene of B/Victoria influenza viruses collected in Guangdong, China from 2019–2022. The same colors in the figure indicate strains from the same year. **B** Phylogenetic analysis of HA sequences of B/Victoria influenza viruses, globally and in Guangdong Province, China. In the region layer, strains from different regions are different colors. Red and blue lines show domestic pre-COVID-19 (before the COVID-19 outbreak in December 2019) and during-COVID-19 (during the COVID-19 outbreak from January 2020 to December 2022) strains in Guangdong, China, respectively

To understand the changes in influenza activity amid the COVID-19 pandemic, we studied the genetic diversity in B/Victoria and A/H3N2 influenza viruses and conducted a phylogenetic analysis of the viral HA and NA genes. HA sequences of influenza B/Victoria strains from Guangdong Province were compared with those sequences from global influenza virus populations before (January to December 2019) and during the COVID-19 pandemic (January 2020 to December 2022). A phylogenetic study revealed that before the COVID-19 pandemic, the B/Victoria virus strains in Guangdong Province were closely related to lineages from diverse areas worldwide. The predominant circulating strain belonged to the V1A.3a evolutionary branch, close to the World Health Organization (WHO)-recommended vaccine strain B/Washington/02/2019 (EPI ISL 341131) for that year. During the COVID-19 pandemic, HA and NA genes of the B/Victoria virus strains evolved into new lineages (Fig. 2A, Fig. S2A). Upon comparison, these strains were close to the local strains circulating in China (e.g., Anhui, Beijing, and Fujian) and to individual lineages originating from Montana, USA, and Sydney, Australia (Fig. S2 A–D). The predominant circulating lineage belonged to the V1A.3a.2 evolutionary branch, which was similar to the WHO-recommended vaccine strains B/Washington/02/2019 (EPI ISL 341131) and B/Washington/02/2019 (21/336) (EPI ISL 7473160) in 2021–2022 (Fig. 2A, Table S4). Thus, during the COVID-19 period, the genetic diversity notably decreased among B/Victoria virus strains in Guangdong Province, with new lineages closely resembling domestic strains within China (Fig. 2B). The HA and NA proteins also underwent non-synonymous mutations (e.g., R147G, N164K, and R290K), suggesting that the B/Victoria viruses were continuously mutating (Table S5, S6).

**Fig. 3:**
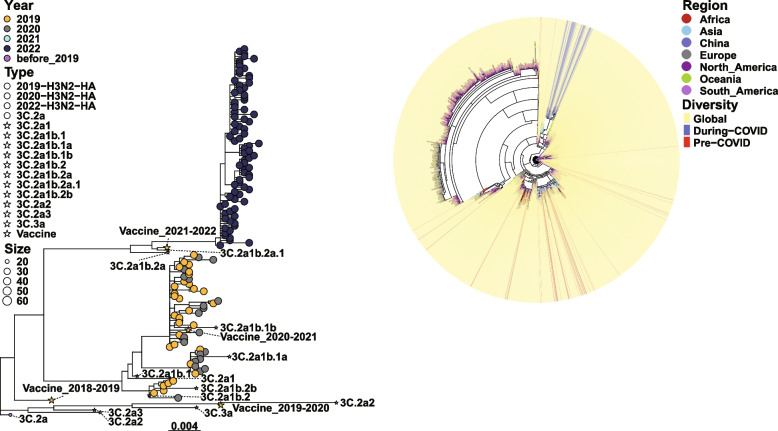
Phylogenic tree of the HA gene of H3N2 influenza viruses during 2019–2022. **A** Phylogenetic tree of the HA gene of H3N2 influenza viruses collected in Guangdong China during 2019–2022. The same colors in the figure indicate strains from the same year. **B** Phylogenetic analysis of HA sequences globally and in Guangdong, China. In the region layer, strains from different regions are different colors. Red and blue lines show domestic pre-COVID-19 and during-COVID-19 strains in Guangdong, China, respectively

Influenza A H3N2 virus exhibited a similar evolutionary trend. Before the COVID-19 pandemic (January to December 2019), three H3N2 sublineages co-circulated in Guangdong. The evolutionary relationships of the HA gene sequences of H3N2 were distributed across multiple regions internationally (e.g., Asia, Africa, Europe, and South America), forming several distinct evolutionary lineages (Fig. S4). During the COVID-19 viral circulation (January 2020 to December 2022), the HA gene of H3N2 viruses evolved into an independent evolutionary branch belonging to the 3C.2a1b.2a.1 evolutionary branch (Fig. S2B). This showed closer genetic proximity to the 2022 WHO-recommended vaccine strain A/Cambodia/e0826360/2020 (Fig. 3A, Table S4) and resembled domestic city lineages, such as those of Chongqing, Guangdong, and Guangxi. Evolutionary analysis of the HA gene suggested that the genetic diversity of influenza H3N2 viruses decreased during the COVID-19 pandemic (Fig. 3B). Although the diversity decreased, multiple mutations, including I29T, I191T, K295E, Y328N, K561N, G604S, Y632F, and I1614M for the HA protein and D1387N and N1394S for the NA protein, occurred in H3N2 viruses during the pandemic compared with those pre-COVID-19. These results indicate ongoing variations in the HA and NA genes of H3N2 influenza viruses during the COVID-19 pandemic (Table S7, S8).

### Evolutionary rates and evolutionary selection pressure of HA and NA genes from H3N2 and B/Victoria in Guangdong, China

**Fig. 4:**
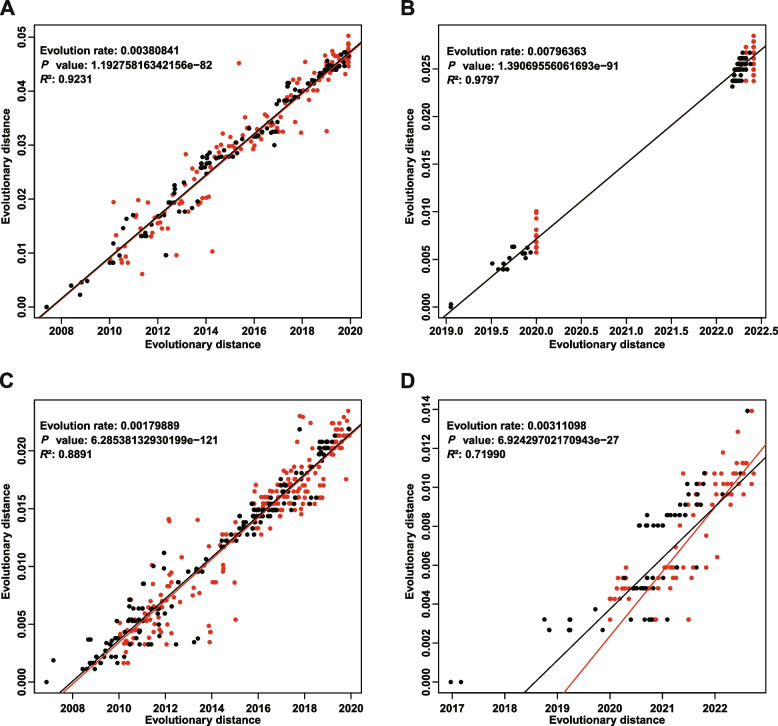
Evolutionary rates of HA genes in H3N2 and B/Victoria influenza viruses before and during the COVID-19 outbreak. **A**–**B** Temporal signal in the HA gene dataset of H3N2 determined by linear regression of root-to-tip distance (y-axis) against sample collection date (x-axis). **C**–**D** Temporal signal in HA gene dataset of B/Victoria determined by linear-regression of root-to-tip distance (y-axis) against sample collection date (x-axis). Left and right represent the evolutionary rates before and during the COVID-19 pandemic, respectively. Red dots represent the evolutionary tree's tips, and black dots represent its internal nodes. The association between root-to-tip distances among strains in different years was determined using regression curve models. *P* value < 0.05 were considered significant

We conducted a detailed analysis of the evolutionary rates of HA genes from H3N2 and B/Victoria in Guangdong, China during 2010–2022 to evaluate the effect of epidemic control measures on the evolution of the HA genes of these viruses. The analysis showed that the estimated HA gene evolutionary rates of H3N2 viruses before and during COVID-19 were 3.80 × 10^−3^ per site per year and 7.96 × 10^−3^ per site per year, respectively (Fig. 4A and B, Table S9); those of the B/Victoria influenza viruses before and during COVID-19 were 1.80 × 10^−3^ per site per year and 3.11 × 10^−3^ per site per year, respectively (Fig. 4C and D, Table S9). The evolutionary rates of NA genes from H3N2 and B/Victoria were shown in Table S9. Evolutionary selection pressure analysis showed that HA and NA genes from both H3N2 and B/Victoria viruses underwent purifying selection pressure (dN/dS < 1), with similar levels of selection pressure on both genes. No loci appeared to be significantly subjected to positive selection pressure acting on H3N2 influenza viruses; however, the HA gene of B/Victoria viruses showed significant positive selection pressure acting on the 264th amino acid site (*P* = 0.0052), suggesting that mutations at this locus may affect the transmissibility of influenza B/Victoria viruses (Table S9). However, no mutation loci for zanamivir and oseltamivir resistance were identified in the NA protein (Table S10, S11).

## Discussion

Influenza viruses are RNA viruses belonging to the family *Orthomyxoviridae* that cause a highly contagious respiratory disease in humans. Both influenza A and B viruses can cause epidemics, with influenza A/H3N2 and B/Victoria being the main strains responsible for current influenza epidemics [19]. However, the COVID-19 pandemic and subsequent implementation of NPIs disrupted the typical seasonality of common respiratory pathogens in many countries [8–10, 20]. This phenomenon also occurred in China, particularly with influenza viruses [21–24]. Despite the substantial decline in influenza activity in Guangdong Province, China, following the emergence of COVID-19, an occult epidemic persisted, and a large seasonal disease outbreak occurred in the summer of 2022 with uncertainty about the origin and timing of the outbreak strain. Therefore, systematic analysis and assessment of the epidemiological and evolutionary characteristics of influenza viruses in Guangdong during the COVID-19 pandemic is imperative.

According to our monitoring data, influenza in Guangdong exhibited biannual peaks before the COVID-19 epidemic. Four influenza peaks occurred in winter 2017–2018, autumn 2018, winter 2018–2019, and winter 2019–2020, consistent with other local studies [25]. But in 2020, as previously reported, the influenza outbreak was effectively contained as the consequence of a significant reduction in people mobilization following the stringent implementation of COVID-19 control measures (such as border inspections, city lockdown, stay-at-home rules, closure of schools and public venues, and suspension of cafeteria dining) [26]. By the end of 2021, influenza cases involving H3N2 and B/Victoria gradually increased again, suggesting that although the influenza epidemic was suppressed for more than a year under the strict control measures, the risk of outbreaks remained even though international travel and tourism had not fully resumed [26]. Our study revealed reduced influenza viral genomic diversity after the easing of COVID-19 restrictions, but occult transmission of influenza could occur under strict NPI measures. Phylogenetic analysis showed that influenza B/Victoria was present in different lineages before the pandemic and widely distributed domestically and internationally. However, the closely related strains of B/Victoria in Guangdong in 2021 (during the COVID-19 pandemic) were mainly converged in China’s domestic branch which belonged to the evolutionary branch V1A.3a.2 and was related to the WHO-recommended vaccine strains B/Washington/02/2019 (EPI ISL 341131) and B/Washington/02/2019 (21/336) (EPI ISL 7473160). Our finding was consistent with that of Huang et al. [27]. These strains were more closely related and had identical antigenicity, suggesting that influenza vaccination may be effective. Influenza virus A/H3N2 resurged in Guangdong during the summer of 2022, had less genetic diversity than pre-COVID-19 H3N2 strains, and was closest to strains from other provinces in China during 2022. The H3N2 strains in the current epidemic in Guangdong belong to subclade 3C.2a1b.2a.1, which is on the same evolutionary branch as the 2022 WHO-recommended vaccine strain (A/Cambodia/e0826360/2020), indicating that the antigenicity of these strains is similar to the vaccine. This phenomenon of decreased genetic diversity has also occurred in countries other than China and in other respiratory viruses. Unprecedented widespread respiratory syncytial virus (RSV) outbreaks occurred in Australia during the spring of 2021. These extended into the summer across two widely separated regions, New South Wales and Australian Capital Territory, revealing a major significance in RSV genetic diversity [28].

Several factors may explain the resurgence of influenza. First, seasonal influenza viruses evolve through antigenic drift to evade pre-existing immunity and gain a competitive advantage by generating new antigenic variants based on their surface protein mutations [29]. Amid the COVID-19 pandemic, HA substitutions I29T, I191T, K295E, Y328N, K561N, G604S, Y632F, and I1614M were found within the major antigenic sites of H3N2 strains, and R147G, N164K, and R290K substitutions were also present in the major antigenic sites of B/Victoria strains, compared with those of pre-epidemic B/Victoria and H3N2 strains. This suggests that the COVID-19 pandemic somehow caused antigenic drift in the H3N2 and B/Victoria strains.

Our results demonstrate that after COVID-19 control measures were relaxed, the rate of B/Victoria HA evolution (3.11 × 10^−3^) was 1.7 times faster than before the pandemic. Likewise, the H3 gene's evolution rate was 7.96 × 10^−3^, which is 2.1 times faster than the strains' pre-COVID-19 evolution rate. We hypothesize that influenza viruses could still be transmitted insidiously despite NPIs being implemented. Once controls were relaxed, H3N2 and B/Victoria viruses evolved rapidly, resulting in an increase in influenza activity. However, the possibility of new introductions of influenza H3N2 and B/Victoria from external sources cannot be ruled out. Evidence suggests that immunity against influenza infection acquired through infection or vaccination wanes gradually, with circulating antibodies beginning to decline within 6 months [30, 31]. During the 3-year period encompassing the COVID-19 pandemic, the population of China lacked exposure to natural infection; therefore, influenza antibodies (either naturally acquired or vaccine-induced) may have decreased or diminished, resulting in an accumulation of susceptible individuals and potentially triggering more severe epidemics [32, 33]. Reduced administration of seasonal influenza vaccinations during the COVID-19 pandemic may have also contributed to increased susceptibility among the population [34, 35]. However, China's low influenza vaccination rates of 3.16% in 2020–2021 and 2.47% in 2021–2022 would have inevitably resulted in a new epidemic. In any case, China's vaccination rates against influenza were 3.16% in 2020–2021 and 2.47% in 2021–2022, respectively; these lower rates will inevitably cause new outbreaks in China [36].” Additionally, not to be overlooked are the decline in the proportion of mask-wearers and the deterioration of self-control consciousness [37]. All of these factors may contribute to the current seasonal influenza outbreak in Guangdong, China.

This study has some limitations. Our data were restricted to virological surveillance data and may have been influenced by biases such as favorable specimen collection and case selection. Moreover, the impact of HA and NA gene mutations on the infectivity and transmissibility of these viruses remains uncertain; additional research is required to confirm their importance.

To curb the COVID-19 pandemic, governments worldwide implemented strict NPIs [38]. However, NPIs do not completely inhibit the spread of influenza in a population. Once domestic and international travel resumes to pre-pandemic levels, influenza viruses will continue to spread unexpectedly [39–41]. Regularly monitoring the evolution of influenza viruses may provide insights and alerts us to potential epidemics in the future.

## Conclusions

The COVID-19-related NPIs disrupt the transmission of seasonal influenza, which may lead to the accumulation of viral mutations and accelerated evolution of H3N2 and B/Victoria viruses. Monitoring the evolution of influenza viruses on a regular basis may provide insights and alerts regarding potential epidemics. To prevent future influenza outbreaks, global preparedness and collaboration are essential.

### Supplementary Information

Supplementary Material 1.

Supplementary Material 2.

## Data Availability

The raw data used in this study can be obtained from the corresponding author upon reasonable request.

## Abbreviations

ARTI: Acute respiratory tract infections
COVID-19: Coronavirus disease 2019
HA: Hemagglutinin
KMD: Kingmed Diagnostics laboratory
NA: Neuraminidase
NPIs: Non-pharmacological interventions
RSV: Respiratory syncytial virus
SARS-CoV-2: Severe acute respiratory syndrome coronavirus 2
WHO: World Health Organization
