# Supplementary material for: Molecular epidemiology and phylogenetic analysis of influenza viruses A (H3N2) and B/Victoria during the COVID-19 pandemic in Guangdong, China
Source: Infect Dis Poverty. 2024 Aug 1;13:56. doi: 10.1186/s40249-024-01218-z (PMC11295596; doi:10.1186/s40249-024-01218-z)
Supplement: Supplementary file 1 — Supplementary Material 1. [file 40249_2024_1218_MOESM1_ESM.docx]

**Table S1. Sequences of primers used to amplify the full hemagglutinin and neuraminidase genes of the influenza A/H3N2 and influenza B/Victoria viruses**

| Target | Sequence (5’>3’) |
| --- | --- |
| H3N2 HA-A | F: TATTGGTCTCAGGGAGCRAAAGCAGGGG |
|  | R: CAATGGGTGCATCTGATCTCATTATTGAGC |
| H3N2 HA-B | F: CCCTAGCAGAATAAGCATCTATTGG |
|  | R: ATATGGTCTCGTATTAGTAGAAACAAGGGTGTTTT |
| H3N2 NA | F: TATTGGTCTCAGGGAGCRAAAGCAGGAGT |
|  | R: ATATGGTCTCGTATTAGTAGAAACAAGGAGTTTTTT |
| Victoria HA | F: AGCAGAAGCAGAGCATTTTCTAATATC |
|  | R: AGTAGTAACAAGAGCATTTTTCAATAAC |
| Victoria NA | F: AGCAGAAGCAGAGC |
|  | R: TGTAGTAACAAGAGCATT |

| **Clinical department** | **Cases** |
| --- | --- |
| Internal Medicine | 56594 |
| Pediatrics | 53050 |
| Hospitalized Department | 16074 |
| Respiratory Medicine | 11328 |
| Intensive Care Unit | 4327 |
| Immunization Room | 3277 |
| Neurology | 1284 |
| Infectious Diseases | 1163 |
| Emergency Medicine | 1150 |
| Pulmonology | 696 |
| Hematology | 541 |
| Surgery | 475 |
| Cardiovascular Medicine | 433 |
| Hematology | 385 |
| Obstetrics | 362 |
| Nephrology | 216 |
| Gynecology | 208 |
| Fever Clinic | 186 |
| Rheumatology | 153 |
| Oncology | 147 |
| Brain Disease | 115 |
| Traditional Chinese Medicine | 104 |
| Others | 2889 |
| NA | 18244 |
| Total | 173401 |

**Table S2.** **Distribution of samples in clinical department**

NA: unrecorded; Others: including organ transplantation surgery, gastroenterology, Geriatrics and so on.

**Table S3. The testing number and the RT-PCR results of respiratory specimens**

|  | Testing number | Positive number | Negative number | A(H1N1)  pdm09 | A(H3N2) | B(Victoria) | Untyped |
| --- | --- | --- | --- | --- | --- | --- | --- |
| Jan-18 | 294 | 85 | 209 | - | - | - | - |
| Feb-18 | 171 | 19 | 152 | - | - | - | - |
| Mar-18 | 258 | 20 | 238 | - | - | - | - |
| Apr-18 | 208 | 1 | 207 | - | - | - | - |
| May-18 | 271 | 7 | 264 | - | - | - | - |
| Jun-18 | 318 | 39 | 279 | - | - | - | - |
| Jul-18 | 92 | 6 | 86 | - | - | - | - |
| Aug-18 | 74 | 2 | 72 | - | - | - | - |
| Sep-18 | 63 | 1 | 62 | - | - | - | - |
| Oct-18 | 50 | 1 | 49 | - | - | - | - |
| Nov-18 | 76 | 1 | 75 | - | - | - | - |
| Dec-18 | 104 | 10 | 94 | 8 | 0 | 0 | 2 |
| Jan-19 | 215 | 16 | 199 | 14 | 0 | 0 | 2 |
| Feb-19 | 16 | 7 | 9 | 7 | 0 | 0 | 0 |
| Mar-19 | 98 | 35 | 63 | 25 | 5 | 2 | 3 |
| Apr-19 | 404 | 79 | 325 | 12 | 9 | 48 | 10 |
| May-19 | 125 | 70 | 55 | 9 | 3 | 52 | 6 |
| Jun-19 | 76 | 53 | 23 | 2 | 3 | 30 | 18 |
| Jul-19 | 54 | 34 | 20 | 1 | 4 | 21 | 8 |
| Aug-19 | 111 | 10 | 101 | 0 | 5 | 2 | 3 |
| Sep-19 | 306 | 4 | 302 | 0 | 1 | 3 | 0 |
| Oct-19 | 1777 | 1 | 1776 | 0 | 0 | 1 | 0 |
| Nov-19 | 74 | 4 | 70 | 0 | 1 | 2 | 1 |
| Dec-19 | 216 | 122 | 94 | 6 | 98 | 14 | 4 |
| Jan-20 | 258 | 123 | 135 | 20 | 87 | 14 | 2 |
| Feb-20 | 207 | 0 | 207 | 0 | 0 | 0 | 0 |
| Mar-20 | 271 | 0 | 271 | 0 | 0 | 0 | 0 |
| Apr-20 | 318 | 0 | 318 | 0 | 0 | 0 | 0 |
| May-20 | 92 | 0 | 92 | 0 | 0 | 0 | 0 |
| Jun-20 | 74 | 0 | 74 | 0 | 0 | 0 | 0 |
| Jul-20 | 63 | 0 | 63 | 0 | 0 | 0 | 0 |
| Aug-20 | 50 | 0 | 50 | 0 | 0 | 0 | 0 |
| Sep-20 | 76 | 0 | 76 | 0 | 0 | 0 | 0 |
| Oct-20 | 294 | 1 | 293 | 0 | 0 | 0 | 1 |
| Nov-20 | 171 | 0 | 171 | 0 | 0 | 0 | 0 |
| Dec-20 | 587 | 2 | 585 | 0 | 0 | 0 | 2 |
| Jan-21 | 435 | 0 | 435 | 0 | 0 | 0 | 0 |
| Feb-21 | 149 | 0 | 149 | 0 | 0 | 0 | 0 |
| Mar-21 | 247 | 0 | 247 | 0 | 0 | 0 | 0 |
| Apr-21 | 365 | 0 | 365 | 0 | 0 | 0 | 0 |
| May-21 | 360 | 0 | 360 | 0 | 0 | 0 | 0 |
| Jun-21 | 358 | 0 | 358 | 0 | 0 | 0 | 0 |
| Jul-21 | 368 | 2 | 366 | 0 | 0 | 2 | 0 |
| Aug-21 | 454 | 0 | 454 | 0 | 0 | 0 | 0 |
| Sep-21 | 701 | 0 | 701 | 0 | 0 | 0 | 0 |
| Oct-21 | 616 | 3 | 613 | 0 | 0 | 2 | 1 |
| Nov-21 | 651 | 15 | 636 | 0 | 0 | 14 | 1 |
| Dec-21 | 789 | 39 | 750 | 0 | 0 | 34 | 5 |
| Jan-22 | 1237 | 21 | 1216 | 0 | 0 | 19 | 2 |
| Feb-22 | 692 | 5 | 687 | 0 | 0 | 3 | 2 |
| Mar-22 | 1573 | 4 | 1569 | 0 | 1 | 2 | 1 |
| Apr-22 | 1030 | 4 | 1026 | 0 | 2 | 1 | 1 |
| May-22 | 1159 | 16 | 1143 | 0 | 15 | 0 | 1 |
| Jun-22 | 2248 | 255 | 1993 | 0 | 241 | 0 | 14 |
| Jul-22 | 3596 | 77 | 3519 | 0 | 74 | 0 | 3 |
| Aug-22 | 1585 | 12 | 1573 | - | - | - | - |
| Sep-22 | 1170 | 1 | 1169 | - | - | - | - |
| Oct-22 | 1201 | 2 | 1199 | - | - | - | - |
| Nov-22 | 1207 | 2 | 1205 | - | - | - | - |
| Dec-22 | 2443 | 5 | 2438 | - | - | - | - |
| Total | 32546 | 1216 | 31330 | 104 | 549 | 266 | 93 |

“-”: Genomic typing was not done.

“Untyped”: The test was not done due to too little sample left.

**Table S4. Antigen epitope mutation site in HA gene of influenza A virus and B/Victoria influenza virus**

| Virus strains | Antigenic epitopes | | | | | | | | | | | | | | | | |
| --- | --- | --- | --- | --- | --- | --- | --- | --- | --- | --- | --- | --- | --- | --- | --- | --- | --- |
|  | A | | B | | | | | | | | C | | D | | E | | |
|  | 140 | 144 | 128 | 159 | 186 | 189 | 190 | 192 | 193 | 198 | 48 | 53 | 121 | 219 | 62 | 81 | 88 |
| A/Hong Kong/2671/2019(2020-2021) | S | A | V | S | N | Q | F | K | L | V | D | T | Y | T | S | T | G |
| A/Cambodia/e0826360/2020(2021-2022) | S | T | V | S | N | Q | F | K | L | V | D | T | Y | T | S | T | G |
| Southern China H3N2 HA(2019) | S | T(4) | V | S | N | Q | F | K | L | V | D | T | Y | T | S | T | G |
| Southern China H3N2 HA(2020) | S | T(1) | V | S | N | Q | F | K | L | V | D | T | Y | T | S | T | G |
| Southern China H3N2 HA(2022) | S | T | V | F(1) | N | Q | F | K | L | V | D | T | Y | T | F(14) | T | G |
|  | 120-loop | | | | | | | 190-helix |  |  |  |  |  |  |  |  |  |
|  | 131 | 132 | 141 | 142 | 144 | 148 | 151 | 215 |  |  |  |  |  |  |  |  |  |
| B/Washington/02/2019 (2020-2021) | H | V | N | A | D | R | E | K |  |  |  |  |  |  |  |  |  |
| B/Washington/02/2019 (21/336) (2021-2022) | H | V | N | A | D | R | E | K |  |  |  |  |  |  |  |  |  |
| Southern China Victoria HA(2019) | N(1) | I(1) | S(1) | T(1) | N(1) | R | K(1) | K |  |  |  |  |  |  |  |  |  |
| Southern China Victoria HA(2021) | H | I(1) | N | T(46） | D | G(48) | E | R(46) |  |  |  |  |  |  |  |  |  |
| Southern China Victoria HA(2022) | H | V | N | T | D | G | E | R |  |  |  |  |  |  |  |  |  |

**Table S5. Mutations in the HA gene of B/Victoria influenza virus before and during the COVID-19 outbreak**

| Position | Base | | Amino acid | | Type |
| --- | --- | --- | --- | --- | --- |
|  | 2019 | 2021-2022 | 2019 | 2021-2022 |  |
| 147 | A | G | R | G | nonsynonymous |
| 164 | C | A | N | K | nonsynonymous |
| 264 | G | A | Q | Q | synonymous |
| 290 | G | A | R | K | nonsynonymous |

**Table S6. Mutations in the NA gene of B/Victoria influenza virus before and during the COVID-19 outbreak**

| Position | Base | | Amino acid | | Type |
| --- | --- | --- | --- | --- | --- |
|  | 2019 | 2021-2022 | 2019 | 2021-2022 |  |
| 291 | T | C | L | L | synonymous |
| 363 | A | T | A | A | synonymous |

**Table S7. Mutations in the HA gene of H3N2 influenza virus before and during the COVID-19 outbreak**

| Position | Base | | Amino acid | | Type |
| --- | --- | --- | --- | --- | --- |
|  | 2019-2020 | 2022 | 2019-2020 | 2022 |  |
| 18 | T | C | A | A | synonymous |
| 29 | T | C | I | T | nonsynonymous |
| 39 | G | T | L | L | synonymous |
| 191 | T | C | I | T | nonsynonymous |
| 295 | A | G | K | E | nonsynonymous |
| 297 | A | G | K | K | synonymous |
| 328 | T | A | Y | N | nonsynonymous |
| 387 | C | A | A | A | synonymous |
| 462 | T | G | A | A | synonymous |
| 489 | C | T | F | F | synonymous |
| 492 | T | C | F | F | synonymous |
| 501 | A | G | L | L | synonymous |
| 561 | G | T | K | N | nonsynonymous |
| 604 | G | A | G | S | nonsynonymous |
| 632 | A | T | Y | F | nonsynonymous |
| 969 | A | G | R | R | synonymous |
| 975 | T | C | V | V | synonymous |
| 987 | T | C | T | T | synonymous |
| 1056 | G | T | A | A | synonymous |
| 1074 | T | A | G | G | synonymous |
| 1323 | G | T | A | A | synonymous |
| 1567 | C | T | L | L | synonymous |
| 1614 | A | G | I | M | nonsynonymous |

**Table S8. Mutations in the NA gene of H3N2 influenza virus before and during the COVID-19 outbreak**

| Position | Base | | Amino acid | | Type |
| --- | --- | --- | --- | --- | --- |
|  | 2019-2020 | 2022 | 2019-2020 | 2022 |  |
| 408 | G | A | Q | Q | synonymous |
| 636 | A | T | V | V | synonymous |
| 957 | A | T | S | S | synonymous |
| 1387 | G | A | D | N | nonsynonymous |
| 1394 | A | G | N | S | nonsynonymous |

**Table S9. Selection pressure and evolution rate of influenza A and B/Victoria before and during COVID-19**

| Gene | global dN/dS | sites under episodic diversifying positive selection | Evolution rate (per site per year) | |
| --- | --- | --- | --- | --- |
|  |  |  | pre-COVID-19 | during-COVID-19 |
| H3N2-HA | 0.2042 | 0 | 0.003808 | 0.007964 |
| H3N2-NA | 0.2159 | 0 | 0.0006511 | 0.0009658 |
| Victoria-HA | 0.21 | 1264, (GAT->GGT)(*P*=0.0052) | 0.001799 | 0.003111 |
| Victoria-NA | 0.23 | 0 | 0.001347 | 0.001468 |

Likelihood ratio testing was used to examine the significance of specific mutation sites under episodic diversifying positive selection. *P*-value < 0.05 were considered significant.

**Table S10. Drug resistance sites in NA genes of H3N2 influenza virus**

| Gene | Position | Amino Acid | Phenotype |
| --- | --- | --- | --- |
| NA | 116 | V | Sensitive to Oseltamivir |
|  |  |  | Sensitive to Zanamivir |
| NA | 119,222 | EI | Sensitive to Oseltamivir |
| NA | 136 | Q | Sensitive to Zanamivir |
| NA | 222 | I | Sensitive to Oseltamivir |
|  |  |  | Sensitive to Zanamivir |
| NA | 274 | H | Sensitive to Oseltamivir |
| NA | 119 | E | Sensitive to Oseltamivir |
| NA | 119 | E | Sensitive to Oseltamivir |
|  |  |  | Sensitive to Zanamivir |
| NA | 198 | D | Sensitive to Oseltamivir |

**Table S11. Drug resistance sites in NA genes of B/Victoria influenza virus**

| Gene | Position | Amino Acid | Phenotype |
| --- | --- | --- | --- |
| NA | 115 | I | Sensitive to Oseltamivir |
| NA | 154 | R | Sensitive to Peramivir |
|  |  |  | Sensitive to Oseltamivir |
|  |  |  | Sensitive to Zanamivir |
| NA | 197 | D | Sensitive to Oseltamivir |
| NA | 276 | E | Sensitive to Oseltamivir |
| NA | 221 | I | Sensitive to Oseltamivir |
|  |  |  | Sensitive to Zanamivir |
| NA | 273 | H | Sensitive to Oseltamivir |
| NA | 117 | E | Sensitive to Oseltamivir |
|  |  |  | Sensitive to Zanamivir |
|  |  |  | Sensitive to Peramivir |
| NA | 117 | E | Sensitive to Zanamivir |
|  |  |  | Sensitive to Oseltamivir |
|  |  |  | Sensitive to Peramivir |
| NA | 294 | N | Sensitive to Oseltamivir |
| NA | 221 | I | Sensitive to Oseltamivir |
| NA | 221 | I | Sensitive to Oseltamivir |

173,401 ARTI patients enrolled

172,389 Were excluded：

1. 140,855 patients have not been recommended influenza testing by physicians
2. 31,330 negative PCR testing of influenza virus
3. 204 outside the range of our study

403 Were assigned to Sanger sequencing;

207 for influenza A(H3N2) positive samples and 196 for influenza B/Victoria positive samples

609 Were excluded

a) 93 untyped

b） 516 Too little sample volume left

1,012 Underwent PCR-based strain typing

**Figure S1. Flowchart of case selection, subtype identification, and exclusion criteria**


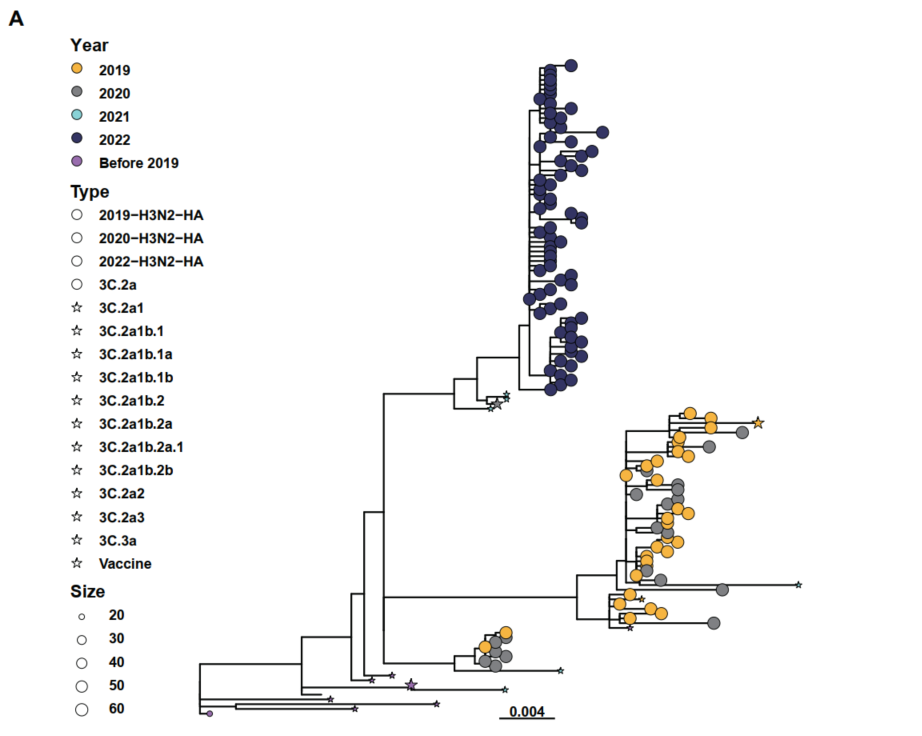


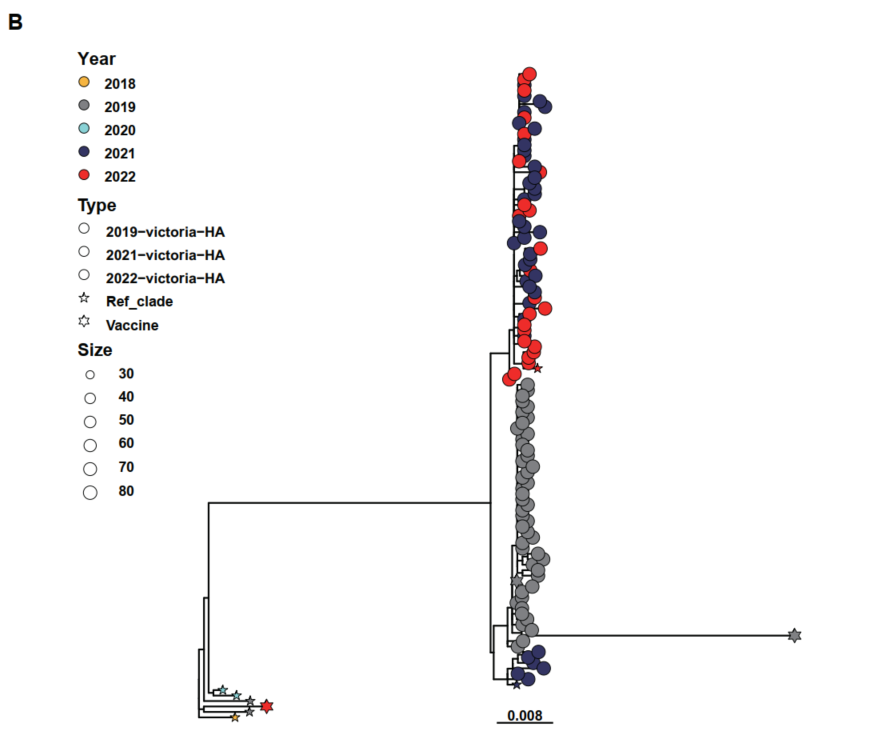


**Figure S2. Phylogenic tree of the NA gene**. A: Phylogenetic tree of NA gene of B/Victoria influenza virus collected in Guangdong Province, China from 2019 to 2022, The same colors in the figure indicate that the strain is from the same year; B: Phylogenetic tree of NA gene of H3N2 influenza virus collected in Guangdong Province, China from 2019 to 2022, The same colors in the figure indicate that the strain is from the same year.


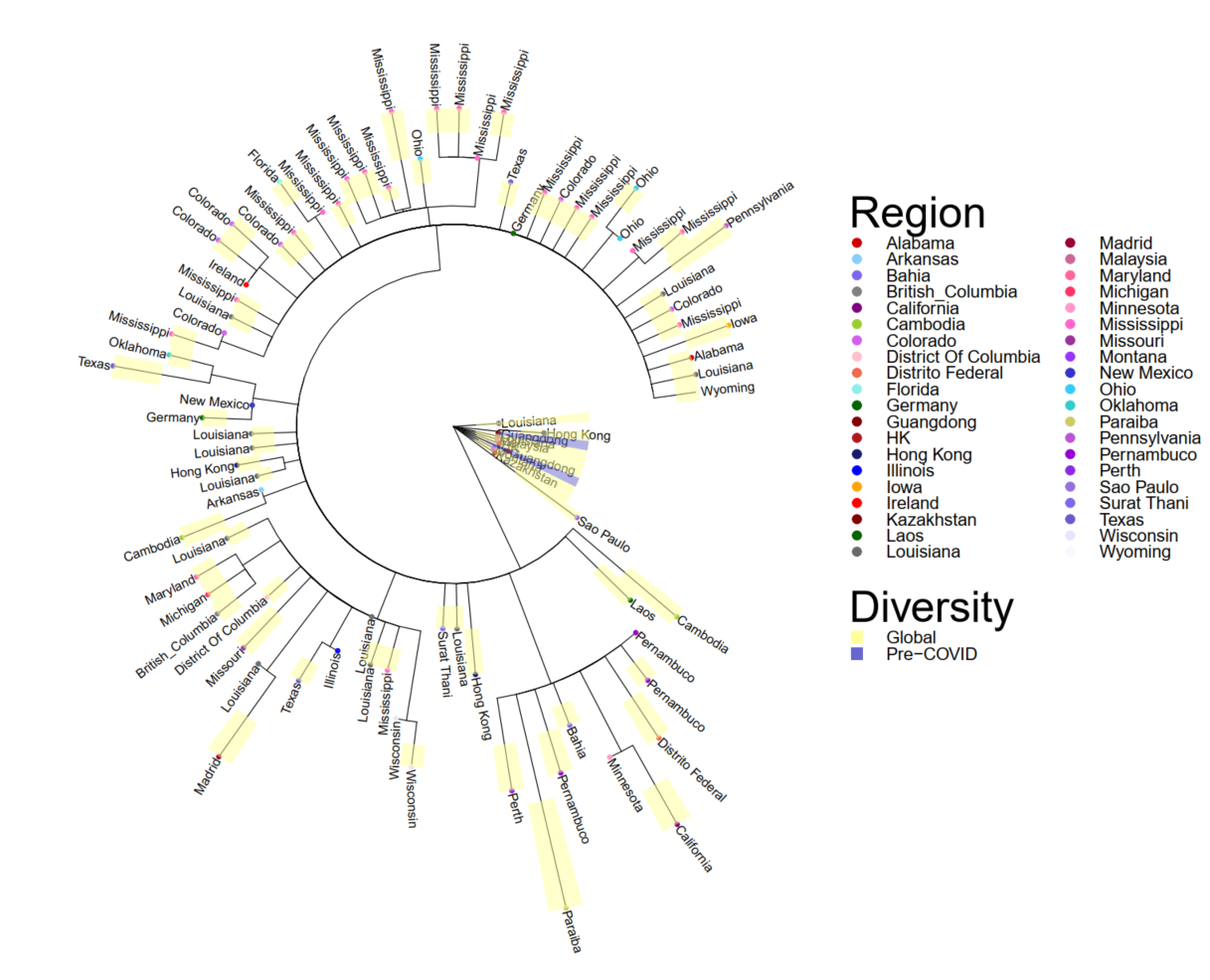
A

B

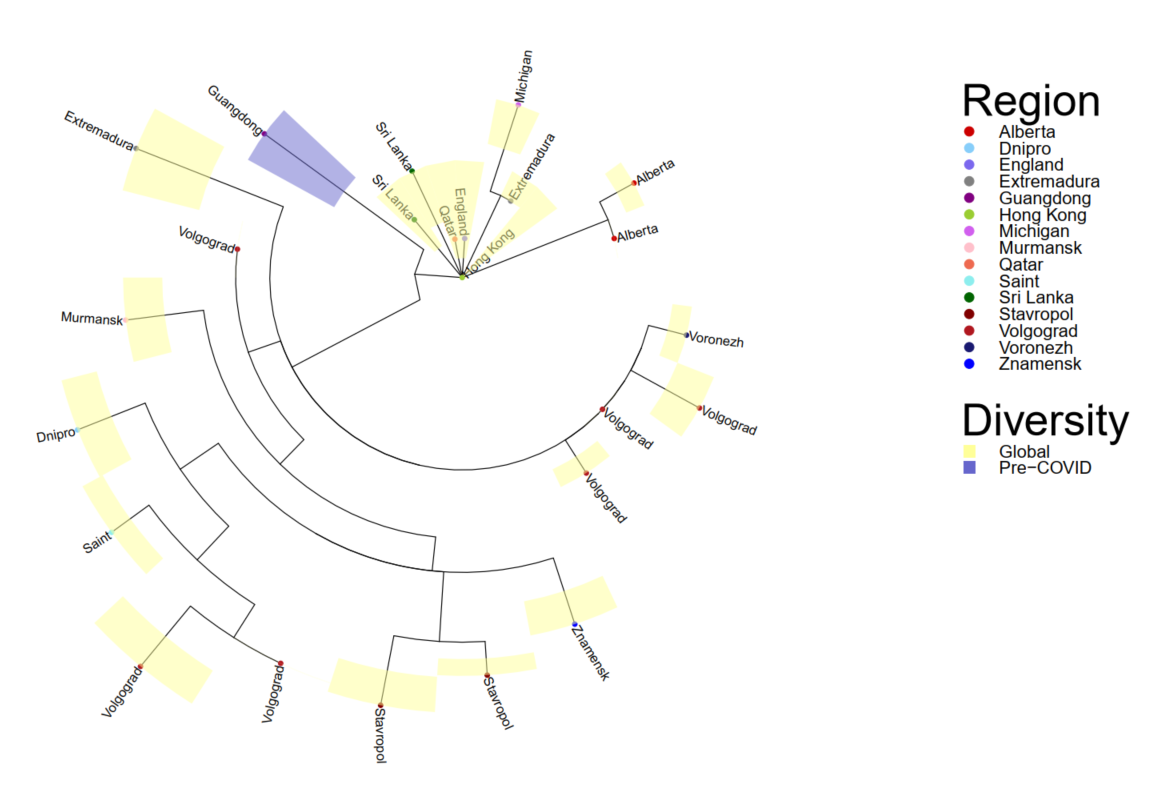


C


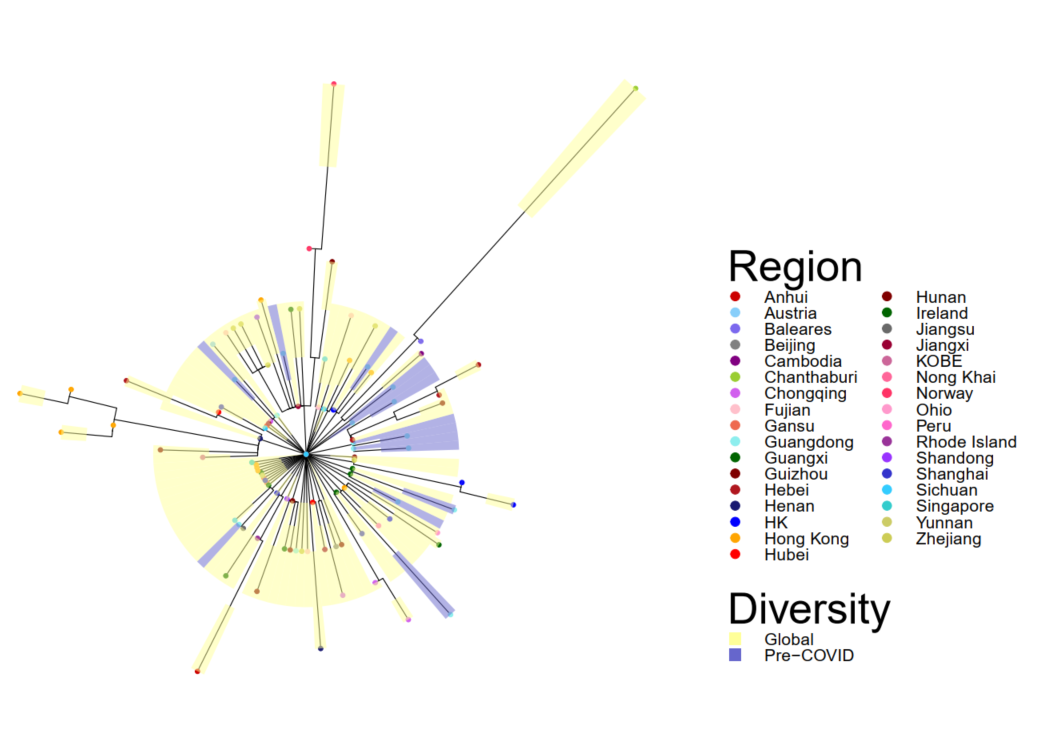


D


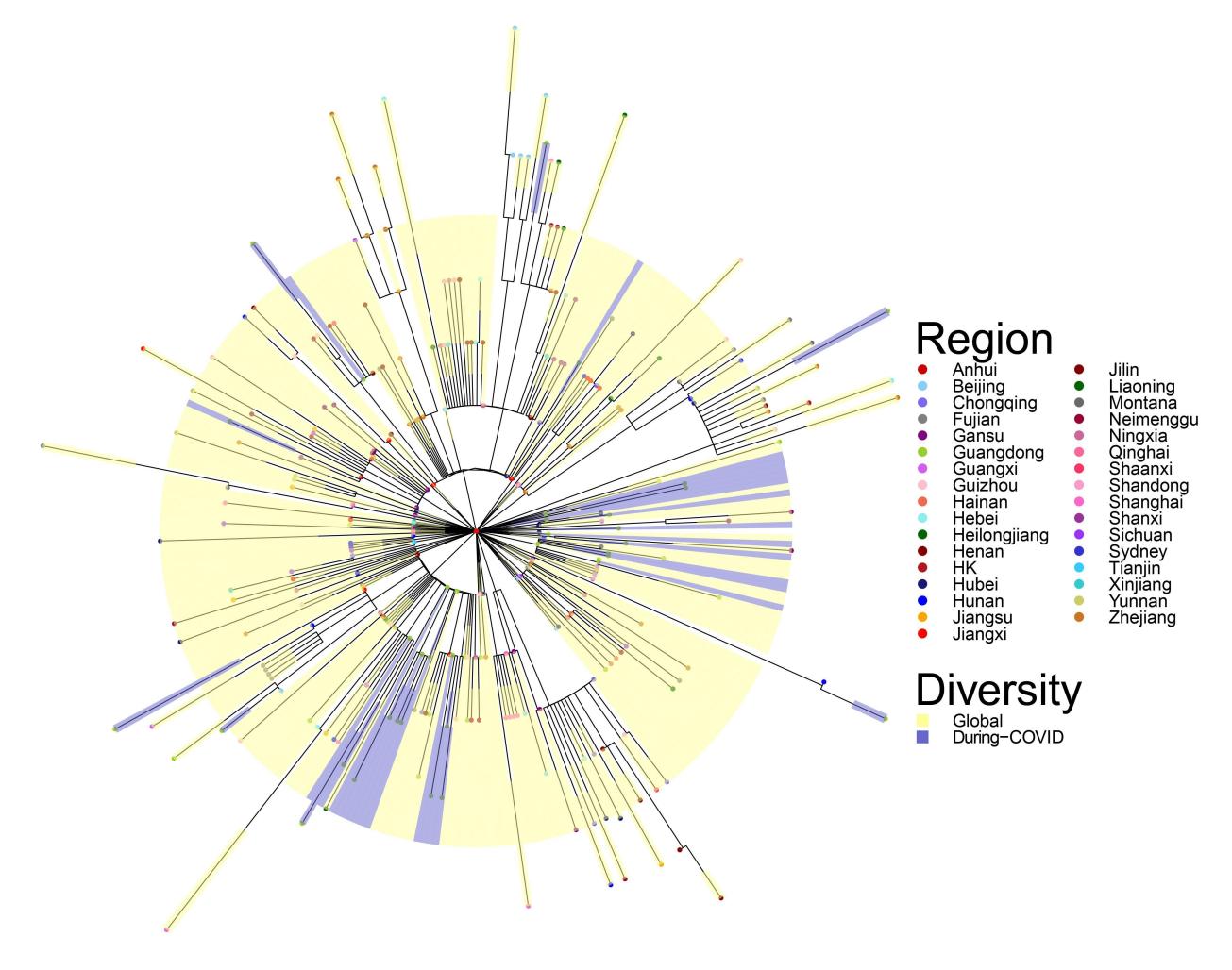


**Figure S3. Phylogenetic analysis of global and Guangdong HA sequences of B/Victoria influenza virus.** The figure shows the sequences of strains in the public database that are most closely related to the evolution of the collected domestic strains. A, B, C: The strains from Guangdong Province, China collected before COVID-19; D: The strains from Guangdong Province, China collected during COVID-19.

Global: Global strains from 2019-2022; Pre-COVID: The strains from Guangdong Province, China collected before COVID-19; During-COVID: The strains from Guangdong Province, China collected during COVID-19.

A


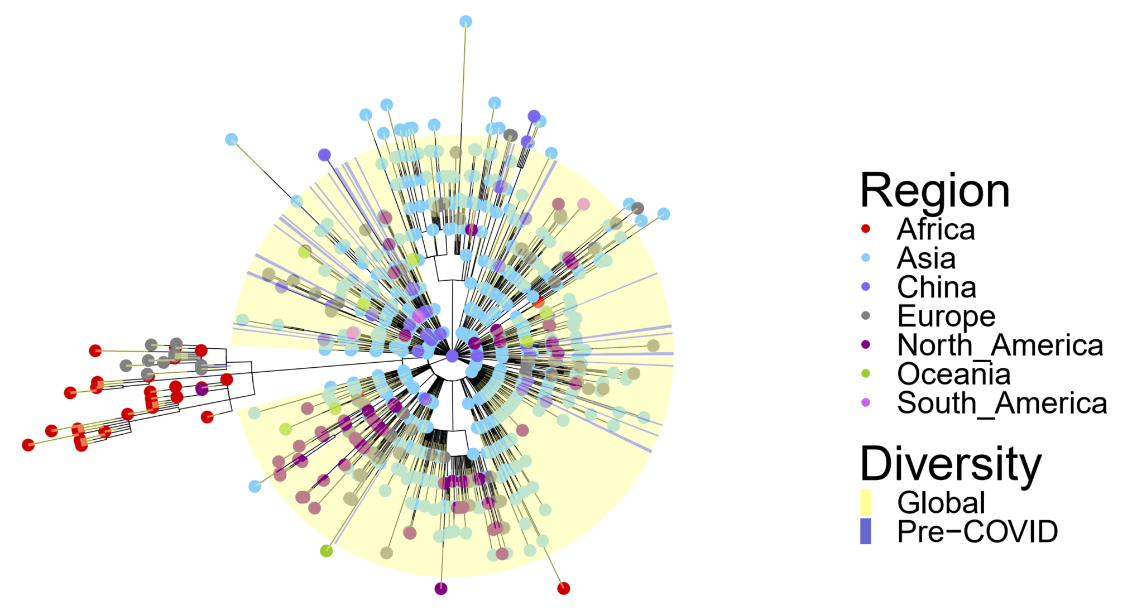


B


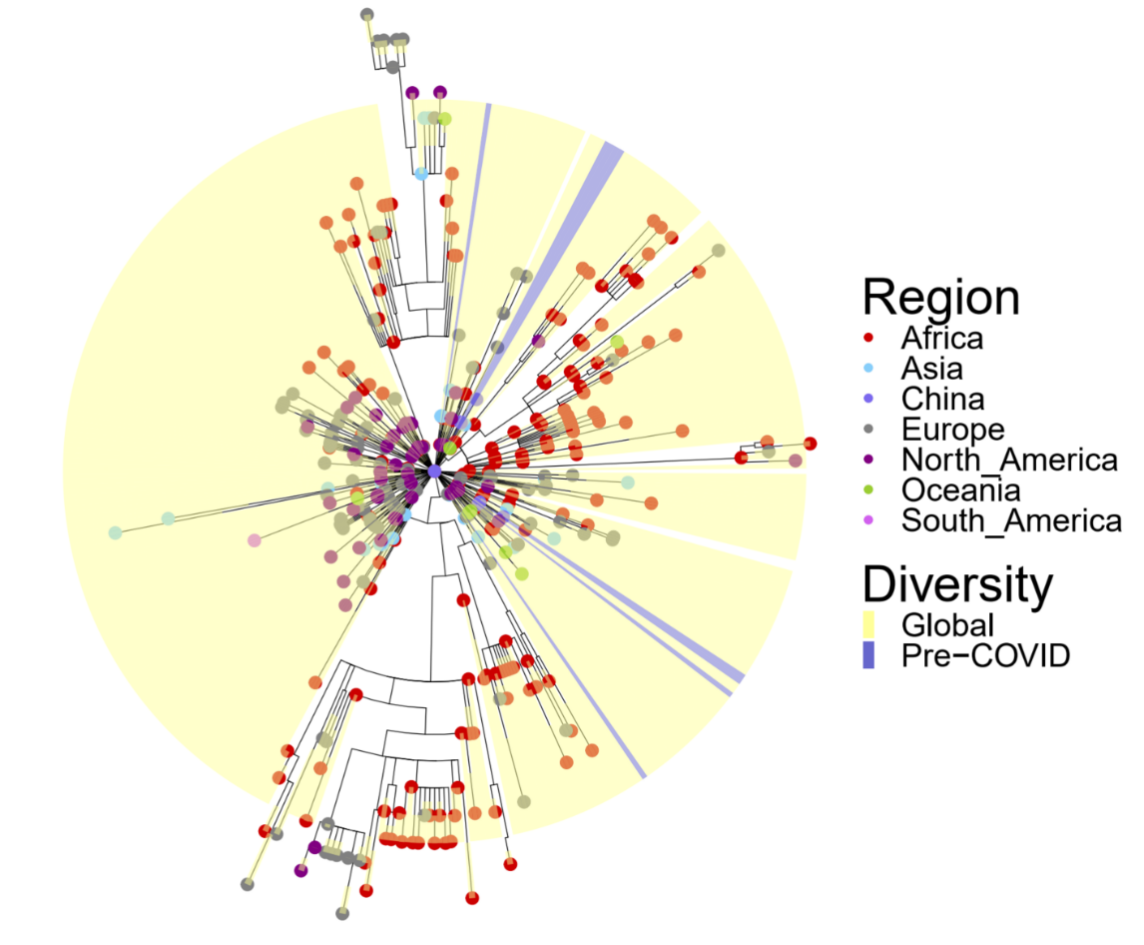


C


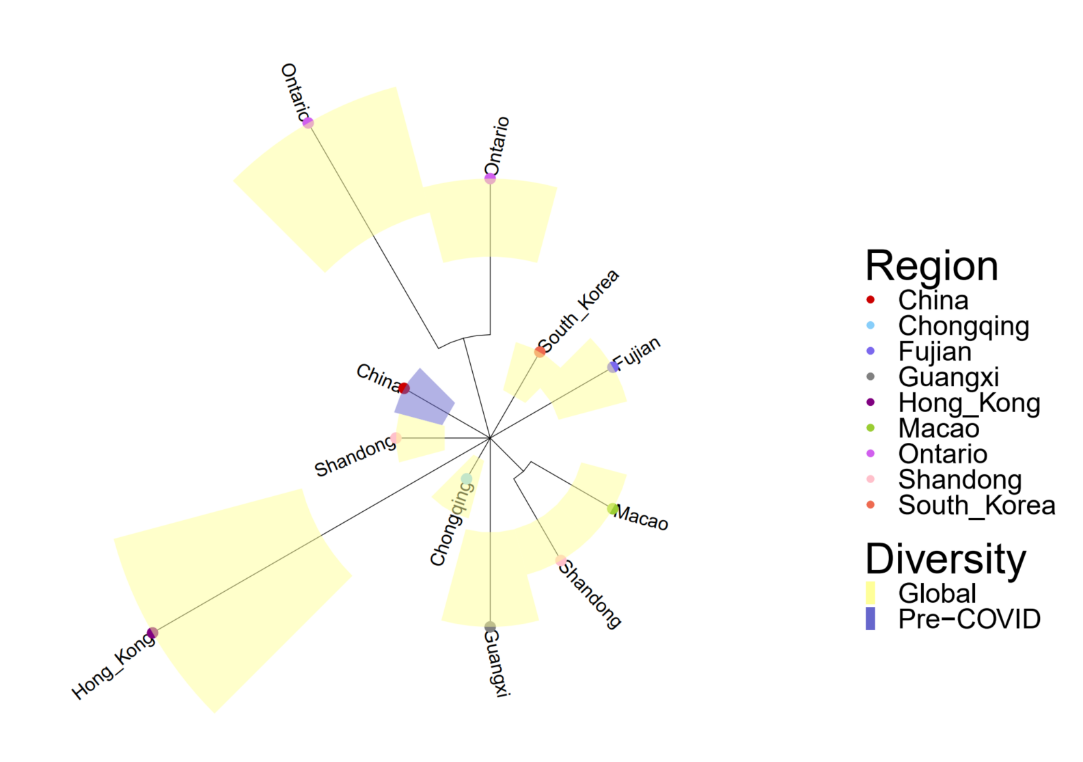


D

**
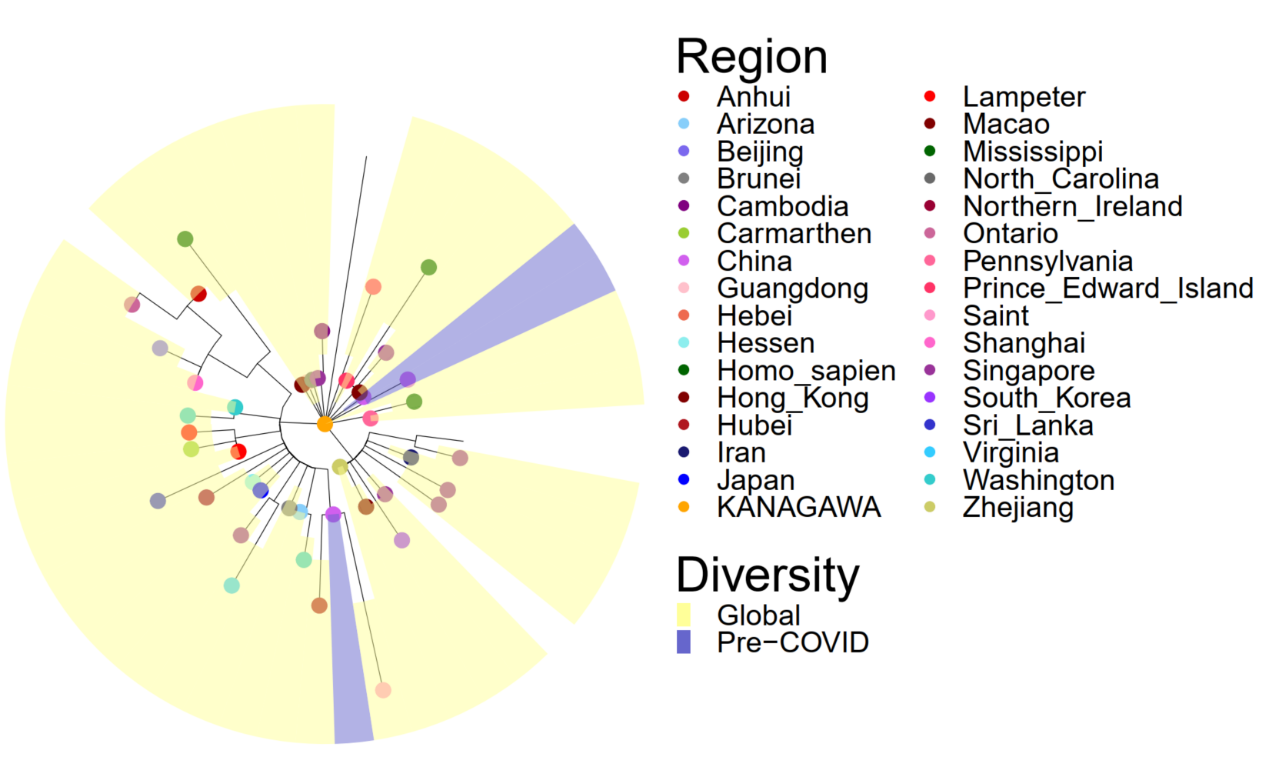
**

E


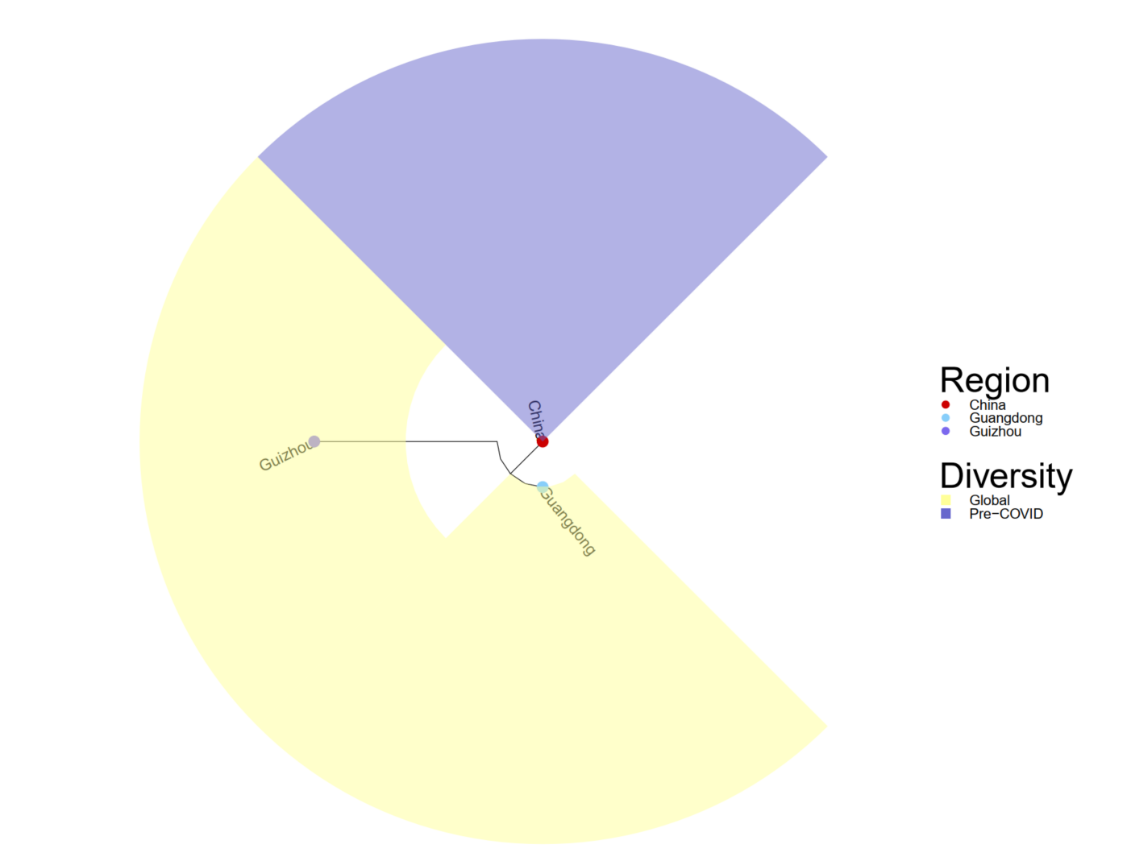


F


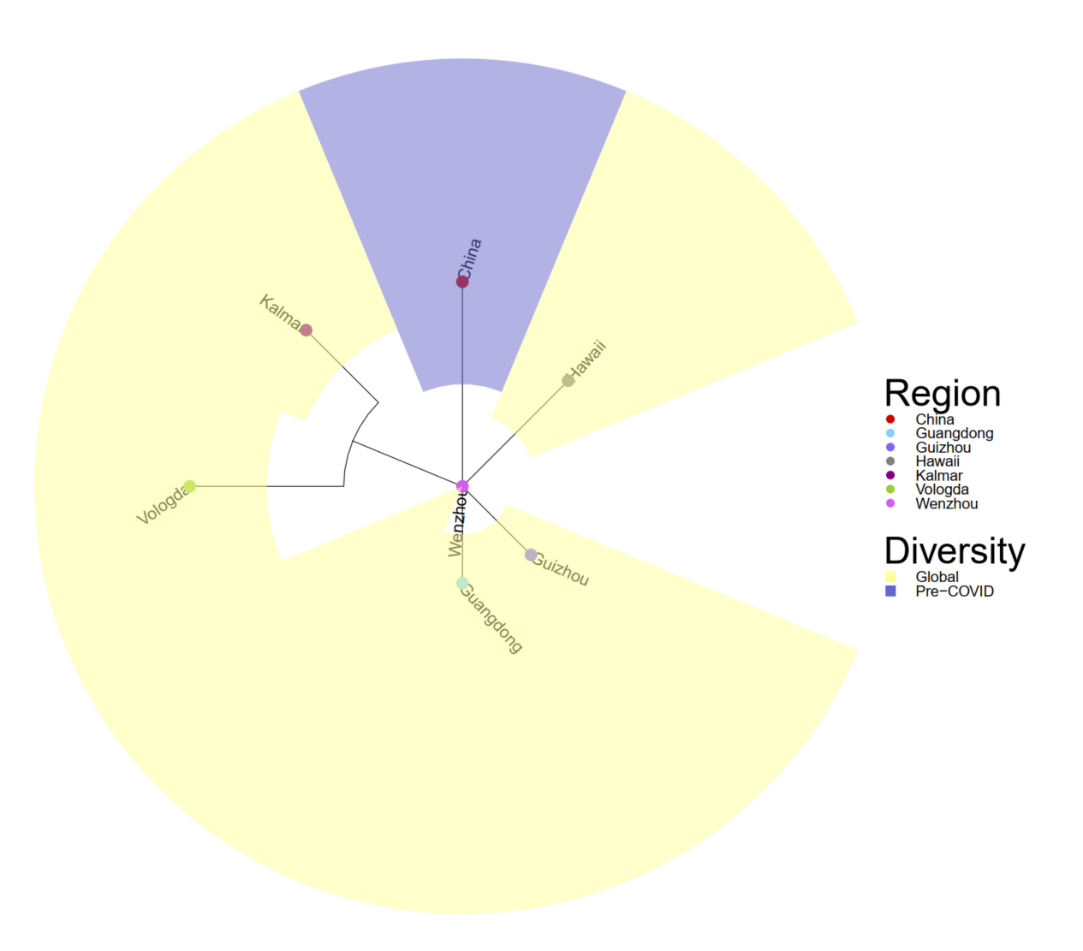


G


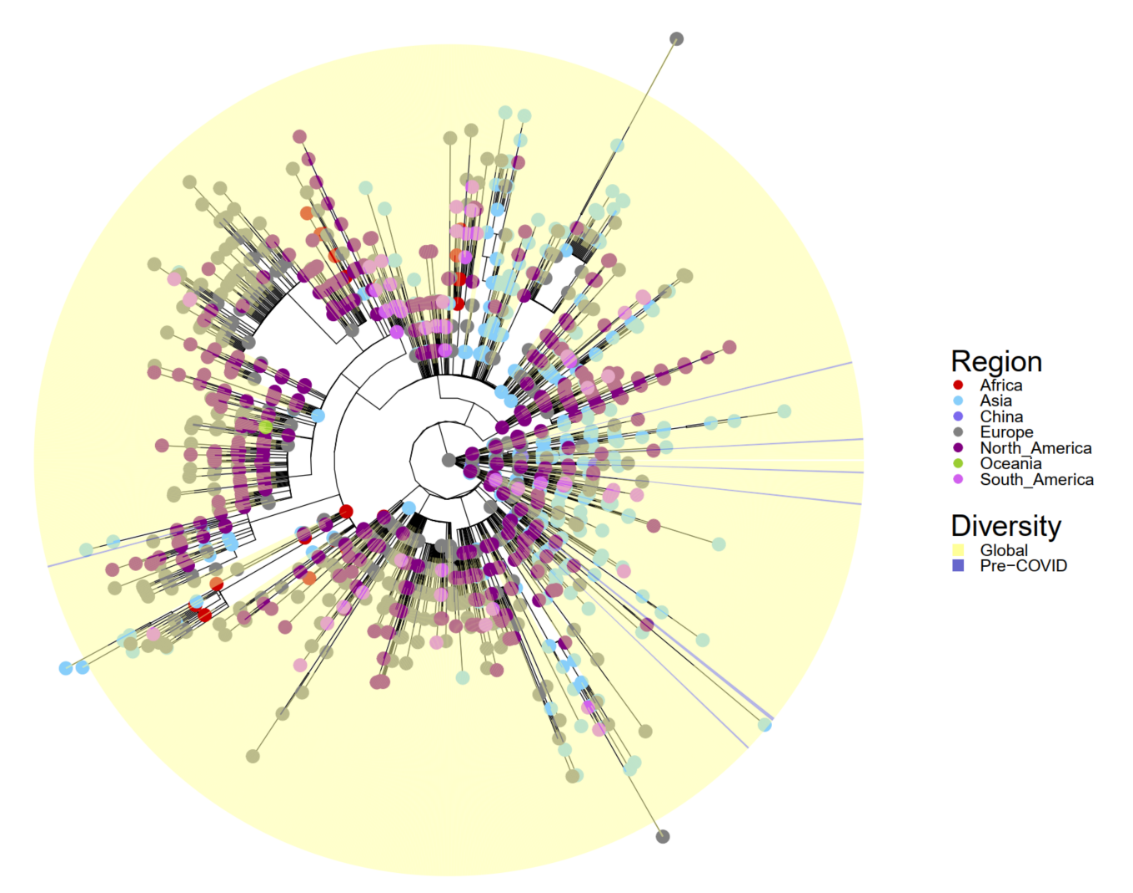


H


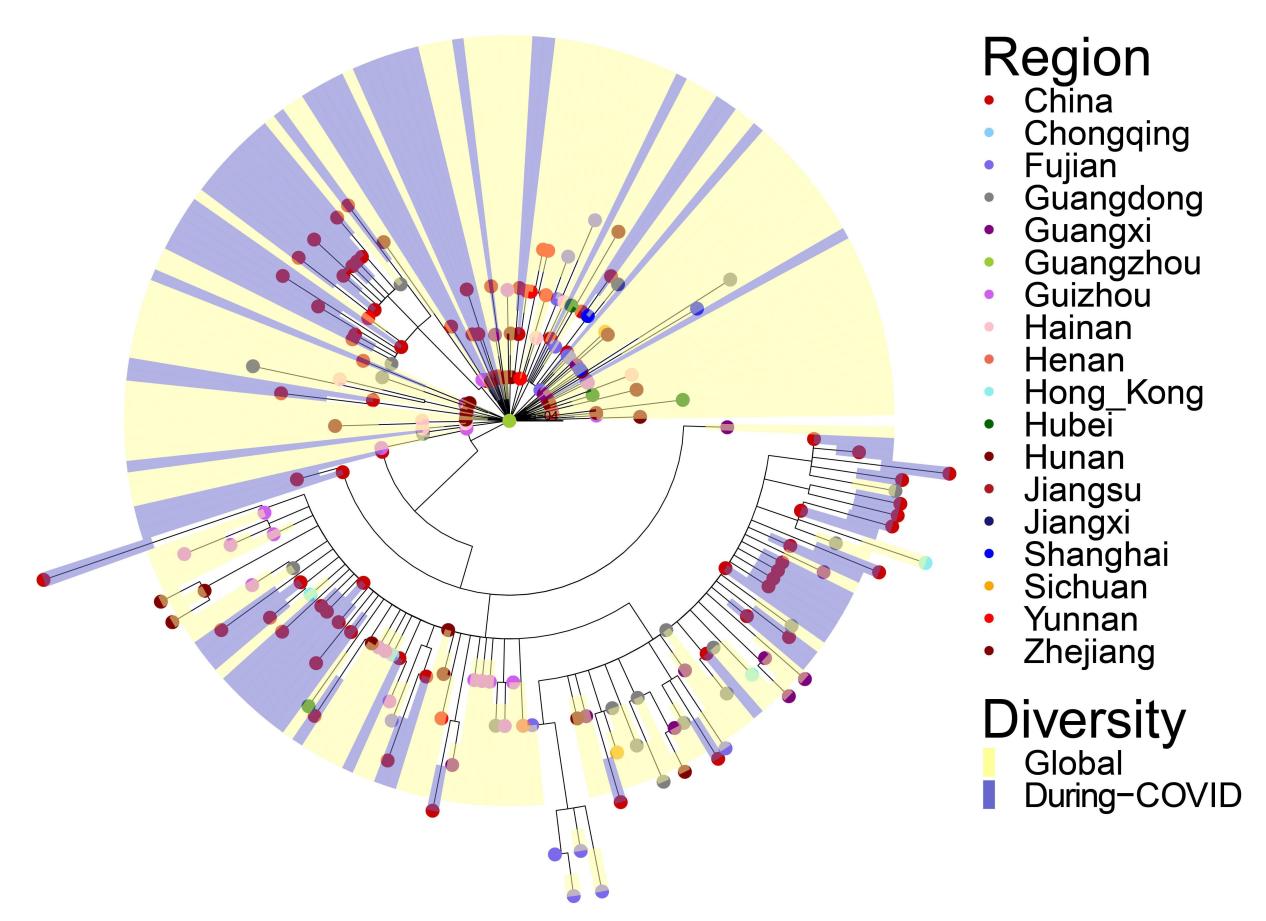


I


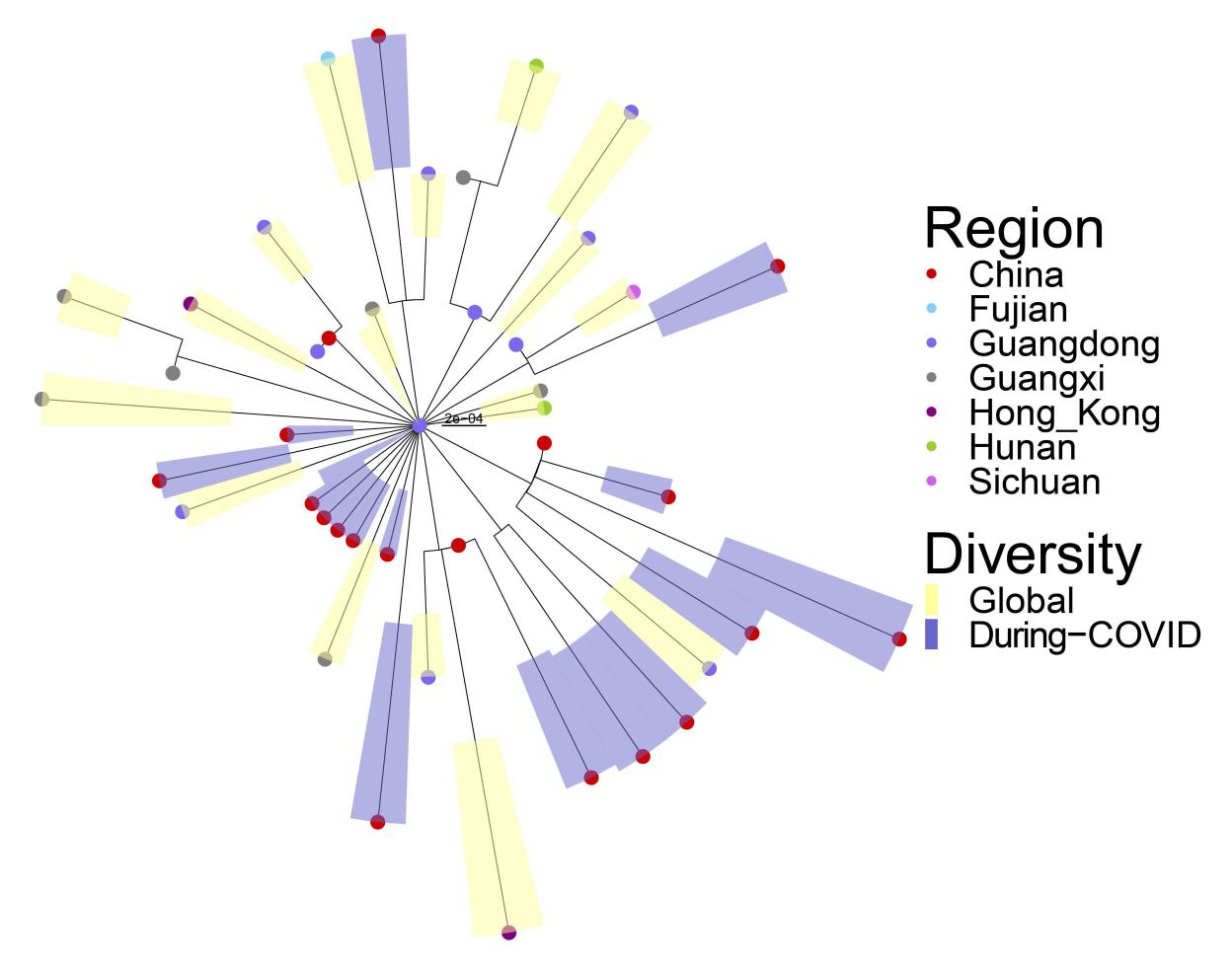


J


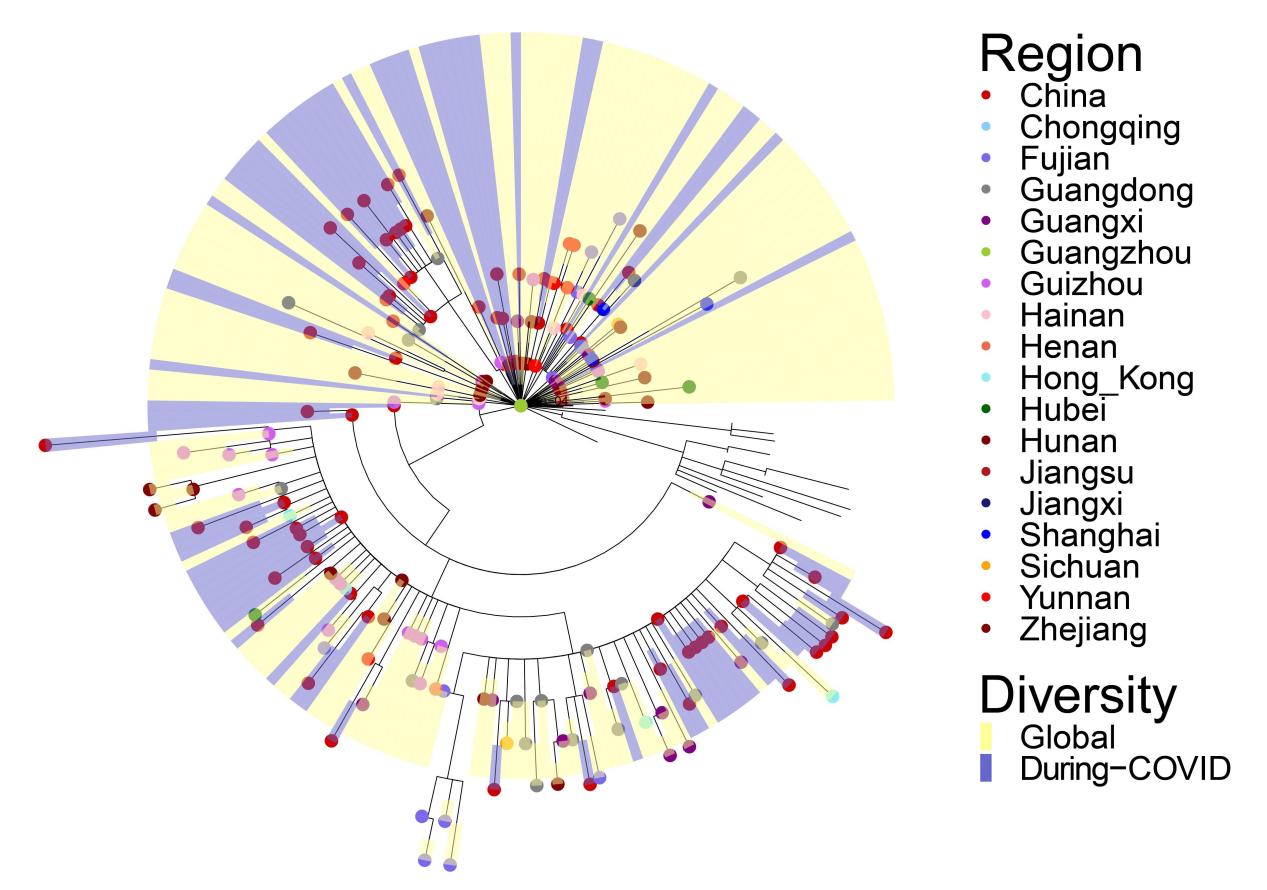


**Figure S4. Phylogenetic analysis of global and Guangdong HA sequences of H3N2 influenza virus**. The figure shows the sequences of strains in the public database that are most closely related to the evolution of the collected domestic strains. A, B, C, D, E, F, G: The strains from Guangdong Province, China collected during COVID-19; H, I, J: The strains from Guangdong Province, China collected before COVID-19;

Global: Global strains from 2019-2022; Pre-COVID: The strains from Guangdong Province, China collected before COVID-19; During-COVID: The strains from Guangdong Province, China collected during COVID-19.
